# Physiology

**Published:** 1836

**Authors:** 


### REVIEWS AND BIBLIOGRAPHICAL NOTICES.

---

### Art. V.—Physiology.

*Animal and Vegetable Physiology, considered with reference to Natural Theology.* By Peter Mark Roget, M. D., Secretary of the Royal Society, Fullerian Professor of Physiology in the Royal Institution of Great Britain, Vice President of the Society of Arts, Fellow of the Royal College of Physicians, consulting Physician to the Queen Charlotte's Lying-in Hospital and to the Northern Dispensatory, etc., etc. Philadelphia: Carey, Lea & Blanchard—1836. 2 vols. pp. 871.

By the Junior Editor.

Among the great variety of physiological works presented to us by late writers, few possess more merit than the one we are about to notice. It is true it contains little or nothing that is new, yet it gives a comprehensive digest of the present state of the science, in a plain and elegant style not often found in works strictly scientific. It is also free from the great exuberance of technical terms which frequently mar the pages of our professional works, especially those of the older writers.

Although Physiology is certainly the basis of a correct Medical Education, yet its study is too exclusively confined to the profession. Elementary works on this department of philosophy should be introduced into our primary schools, and their study as much enjoined upon the pupils, as that of the various books on natural, moral, or mental philosophy. Why should man remain ignorant of himself while he pushes his researches into the structure and modes of action of that which surrounds him? Nothing, perhaps, would more enable him to resist the effects of noxious agents, or to preserve himself from the swarms of empirics which infest both city and country. Although this work is not designed for the school-boy, still we are glad to find it in the hands of the extra-professional reader. As it is written with a special reference to Natural Theology, it will doubtless be useful wherever found, either as a theological or philosophical work.

Our author commences with an introduction on the final causes of organic action. Here he notices the relations existing among the various phenomena of living matter, of which the mind has cognizance. The laws of nature are nothing more than the uniformity, which takes place in the succession of events, and can only be discovered by a careful study of the circumstances, which regulate the phenomena of nature, whether vital or physical. Nature is every where connected, either by cause and effect, or means and end, and the unknown links which unite both are termed the powers of nature. In mechanical philosophy, these powers are called forces. It is a *law* of nature, that where a body is projected from the earth, it will immediately return to its surface, unless sustained by an opposing agent, and attraction is the controlling *force*. It is also a law of nature, that organic action must cease without a circulation of fluids, and the powers which accomplish this circulation, reside in the various tissues, of which the organ is composed.

Our author occasionally advances doctrines, similar to those of the older philosophers. In the introduction, which we have just mentioned, he affirms that the embryo of an organic being, contains within itself, the rudiments of the future vegetable or animal structure, and that the process of nutrition does nothing more than fill up the outlines, already sketched on the living canvass. We think that the best microscopic observations, will not confirm this doctrine, for although the embryo contains the rudiments, or rather the elements of the future animal, still the most imperfect outlines can never be traced in the ovum. It is made up of a homogeneous mass, differing in the various species, according to the place the parent occupies in the scale of organization. Darwin, in the heat of his poetic fancy, advocated the doctrine our author contends for, but his followers were forced to abandon it in this particular at least. Perhaps no subject in physiology is more involved in obscurity than that of nutrition, and it is especially so when we refer it to the primary formation of the animal or plant. We are aware that every animal, however exalted, springs from a given point, a mere atom of matter, but the refined power which draws other material particles to its aid, and endows them with a kind of associate or instinctive feeling, which causes them to unite, until the assemblage is complete, and the entire mass is endowed with sensation, motion, and intelligence, will probably remain forever in obscurity, or be revealed only to finer sensibilities than those of man.

In the first volume, Dr. Roget treats, especially, of the mechanical functions concerned in the process of organization. He commences with the lowest order of life, and traces the peculiarities of each, through all the genera and species of plants and animals, until he arrives at man himself. His chapters on the organization and development of vegetables, are among the most valuable portions of the work. This department of physiology, is too much neglected by the writers of the present period. Few, even among medical philosophers, study the peculiarities of vegetable organization, although it constitutes the basis of a sound physiological education.

The principal parts of the plant are the root, stem, and external covering.

The most elementary texture of vegetables, consists in transparent vessicles of a circular or rather oval form, arranged in an irregular order, and connected together by the walls of the cells. These cells, however, vary both in shape and size, in different specimens, and at different periods in the same specimen. Sometimes they are round, at others they appear like hexagonal prisms resembling the cells of honey-comb. Their diameters also vary from the 30th to the 1000th of an inch, but the medium size is about 1-12th of a line, or 150th part of an inch. Some writers have attempted to class these cells according to their shapes and sizes in different plants, but such classifications can never throw any light on vegetable physiology.

It is a disputed point whether the cells are open or closed, but the truth cannot be easily ascertained, for although they are sometimes filled with fluids, the microscope has failed to reveal to us the passage through which they enter. It is more than probable that they pass through the walls by means of the endosmosis of Dutrochet. As the cells are circular, oval, or prismatic, there must necessarily be a number of intercellular spaces existing between them, which perform an important part in the nutrition of vegetables.

The fluids in both the cells and intercellular spaces differ essentially in the arrangement of their proximate elements. In some instances they are filled with a simple watery fluid, in others with a vegetable secretion, and in others with air alone. The sap of vegetables, unlike the blood of animals, contains some of the proximate elements of the plant in a visible form, of which fecula is the most common and easiest discovered. We have before us the stem of a plant in which we can see, by the aid of a good lens, particles of a dark color, completely detached from the walls of the cells, but whether they are fecula, lignin, or some other agent, we cannot possibly determine. Our author observes, that the coloring matter of leaves and flowers can frequently be detected in their vessels, another proof that the salts, etc., of the body may exist in the blood, uncombined with other agents.

The vessels of plants are various. Sometimes they form spiral tubes, at others they are mere continuous capillaries, and at others they are open vessicles, connected together like rows of beads. In some instances they contain fluids, but mostly air; which is of as much importance in vegetable as animal life. In some plants, especially of the aquatic kind, the air-cells are so large that they contribute much to their buoyancy.

Besides cells and vessels, plants contain a number of other organs, such as glands, which separate the resinous and other productions of plants from the fluids which enter into their structures. But time will not permit us to enter into a more minute analysis of our author's chapter on vegetable organization. It is of much interest to the student of nature, and one that contains more on that subject, than can be found in three times the number of pages in any work with which we are acquainted.

In relation to the development of vegetables, it appears that the growth takes place in two modes; the one by additions to the external parts of the plant, the other by successive growths from the internal surface. The former is termed *Exogenous*, and the latter *Endogenous* plants. The first embraces the oak, elm, beech, and buckeye, together with the great proportion of our northern trees and shrubs, while the last includes the principal part of the growth of southern climates, such as the Date, and Cocoa-nut tree, together with the sugar-cane, and other similar vegetables.

The endogenous growth, of which the palm tree affords a good example, is the most simple. The stem extends to a great heighth, and instead of dividing into branches, terminates abruptly in a tuft of leaves. The first stage of the growth consists in a circle of leaves which shoot upwards from the neck of the plant, and attain during the first year a certain size. The following year another circle of leaves arises, but they grow from the interior of the former circle, which they force outwards as the vegetation advances, and as ligneous matter is deposited. When the external portion of the stem becomes sufficiently hard to resist the pressure from within, the central layers are pushed upwards, and the plant is annually elongated. Decandolle compares this development to the drawing out of the sliding tubes of a telescope. At the close of the season the leaves fall off, and leave the traces of their former existence, by a circular impression upon the stem. The age of the tree may readily be determined by the number of these circles or knobs. Endogenous plants have neither bark, pith, nor concentric layers.

The structure of exogenous trees, is much more complicated, for when fully grown they are composed of wood and bark, which is again subdivided into various parts. The wood consists of *pith* or medulla, harder wood, surrounding the pith in concentric layers, and external softer wood, or *alburnum*, which is also formed in concentric growths on the outside of the former. The bark is divided into the *Liber* and *cellular envelope;* the external part of which is termed *Epidermis.* All of these various layers, together with the *medullary rays* radiating from the pith to the circumference, may readily be distinguished by a horizontal section of the stem.

Every vegetable stem, or every branch which arises from it, springs from a germ or bud of inconceivable minuteness, totally inappreciable by the finest lenses. When large enough to be discovered it consists of the various parts of which the plant is composed, folded up in the smallest possible circumference. The first year is occupied in woody plants, by the formation of pith, first layer of wood, and bark with which it is covered. During the succeeding years, the layers are annually formed on the outside of the wood, and inner portion of the bark, until the growth is complete.

The spiral growth of vegetables, forms one of their most singular features. It is more or less observable in every tree and plant, but especially in the vines, where it seems to be essentially necessary, enabling them to ascend the various objects within their reach, for the purposes of support and nourishment. It has been supposed by many ingenious philosophers, that this was the result of the action of the sun, on the plant, during the early period of its growth, but this supposition our author thinks cannot be supported.

The roots of plants perform the same offices in the vegetable, that the lacteals do in the animal. They select the food from the soil, and convey it into the structure of the plant, by means of a capillary circulation. It is also more than probable, that they return a portion of the sap and vegetable excretion to the earth, after the sap has performed its various offices in the economy of the plants.

Besides the parts already mentioned, plants are furnished by a great variety of organs, of but little importance in their development. They appear in many instances to be designed as instruments of defence. The sting of the nettle bears a striking analogy in its structure, to the poisonous fangs of serpents.

Our author proceeds, in the next place, to notice the organzation of animal structures. Here he mentions the various tissues of which the animal is composed, and gives a detail of the properties of each, as well as the manner in which they are arranged in the organs of the body. In speaking of the muscular tissue, he observes, that the living body contains within itself, a principle of motion not referable, as far as we can perceive, to any of the primary forces which exist in the inanimate world. This principle has been termed *contractility.* In animals of the simplest structure, every part of the substance of the body seems to be equally endowed with this contractile property, although exhibiting no appearances of a fibrous structure. This is the case with all the zoophytes, such as the *Infusoria*, *Polypi*, *Medusæ*, and the simpler kinds of *Entozoa.* Here our author seems to think that a fibrous structure is not necessary in order to secure animal contractility; a doctrine at variance with the notions of some of the older physiologists. As a means of motion, however, contractility resides principally in the muscular tissue, even in the lower order of animals. In these the muscles are not always attached to bones, but frequently adhere to the external integument which increases the mobility of the animal.

He observes that there exists no traces of muscular contractility, in any of the vegetable tissues, but this assertion seems to be opposed by many of the phenomena of plants, for some vegetables evidently possess considerable motion in their branches and leaves, when irritated by external agents.* It is true this motion may vary essentially from that produced by muscular contractility, yet the resemblance between them must forcibly strike the mind of the observer. The remainder of the chapter on muscular motion, though well written, possesses but little that is new or interesting, except to those who have not studied the subject. His positions are the same with those of Bostock, Majendie, and other standard writers on physiology.

The next chapter is one of interest, and contains much that is new to those who have never studied comparative anatomy. It treats of the mechanical functions in *Zoophytes;* the first departure from mere vegetable life in the scale of animal organization. Nothing can be more interesting to the philosopher, than to trace the various links which bind the organic world into one continuous whole. The class of animals which our philosophic author is about to notice, is so intimately associated in its mechanical functions, with that which we have just passed, that it is still a disputed point as to the propriety of separating them. They, however, doubtless form distinct departments in nature, and should be studied as such, although they have much in their arrangement common to both.

The most simple form of animal life is confined to the water, where it is constantly supported by a hydrostatic pressure, almost equal to the attraction of gravity; hence the simplicity of its mechanical structure, an erect position being altogether unnecessary. Some of the Zoophytes or animated plants, are equally as destitute of motion as the vegetable, being fixed by a kind of pedicle, to the rocks, upon which they are placed. The *Porifera*, or sponges, a class of animals abounding in every part of the ocean, from the shores of Greenland to the coasts of Australia, are of this species. In their general appearance, they resemble plants, but their internal

* The leaves of the *Mimosa pudica*, and the *Dionaea muscipulata* move with facility when irritated. The same is true of the leaves and stamina of many other plants.

organization differs from any vegetable structure. They are composed of a soft flesh, intermixed with solid and tubular fibres. The solid portions are horny, and contain large quantities of nitrogen, an element seldom found in vegetables, together with a kind of silicious or calcareous deposit. This fibrous tissue may be said to form the skeleton of the animal, for the fleshy portion is so tender, that the slightest force is sufficient to tear it asunder, and thus permit the fluids to escape. When examined by the microscope, the soft flesh is found to contain a great number of minute grains, suspended in a transparent jelly. This gelatinous substance was said, by the older writers, to contract upon the application of irritants, but our author thinks this opinion unsustained by recent observations. With due deference, however, to the high authority which he quotes, we must still think that sponges contract upon the application of their peculiar stimuli. We cannot otherwise explain the currents of water which are constantly issuing from their interior, for the animals no sooner die than the streams cease. Roget thinks that the currents are occasioned by innumberable fibrils, or *cilia*, placed within the interior canals, but he acknowledges that these cannot be detected even by the aid of the best microscopes. We, however, think it more probable, as well as more philosophic, to consider these living fountains as the offspring of a peculiar animal contraction, especially as the impetuous torrent is the means of furnishing the animal with its usual quantum of food.

Sponges are propagated by means of *ova*, or small buds without envelopes, which are thrown off from the body of the parent at different periods. At first they are loose, and float in various directions through the medium, in which they are placed, until they are attached to the rocks, when they commence their growth. Our author mentions many singular circumstances in relation to the growth of the sponge, which we have not time to enumerate. They all prove it to be a regularly organized animal, with habits and instincts peculiar to itself.

Passing by the remarks of our author on *Polypifera*, a species of animals differing but little from sponges, we come to his observations on the *Infusoria*, a family of animals imperceptible to the naked eye. Unlike the order already mentioned, these minute beings are not fixed to any particular spot, but are found in multitudes in all infusions of animal or vegetable matter, which have been kept for a sufficient time. Their forms can only be, in general, detected by the microscope, although some of them are sufficiently large to be visible to the naked eye. The infusoria, however, are divided into a great variety of species; some of them so extremely small, that a single drop of water contains nearly five hundred millions; a number equal to that of all the human race, yet these living atoms appear to be in the possession of habits and feelings, peculiar to themselves! Indeed professor Ehrenberg, has recently found that they are furnished with internal cavities, for the reception of food, and he thinks it probable that they are also endowed with a muscular, a nervous, and a vascular system. Some suppose that the contagion of small-pox, measles and other specific diseases, is composed of minute animated beings, similar to those we have just mentioned. An ingenious paper on this subject, may be found in the last number of our Journal, by Dr. Riddell.

The infusoria vary much in shape as well as size. Sometimes they are cylindrical, like the *vibrio*, or vinegar eel, at others they are round, flat, stellated, or horny like the *Volvox globator*, or *Proteus*.

A great variety of other animals belonging to the Zoophyte family, are investigated in the chapters following those we have mentioned. We shall, however, be compelled to pass them by for the present, in order to notice what is said on the *Mollusca*.

Although this class is not necessarily fixed, still it embraces but few species that are endowed with the power of locomotion. It is true that many bivalves change their places, but this is accomplished by rapidly closing their shells, by means of a muscular apparatus, which gives an impetus to the animal, by forcibly expelling the water contained within its calcareous coverings. The structure by which this is accomplished, is the principal part of the chapter we shall notice. The valves of the shell of the muscle or oyster, for instance, are united at the back by means of a hinge joint, secured by a strong ligament, which is partly fibrous and partly cartilaginous. The cartilaginous portion is extremely elastic, and is placed within the joint, so that when the shell is closed it is considerably compressed. In this situation its elasticity contributes much towards the opening of the valves, by which means the water passes both to and from the animal, with perfect freedom. As it is necessary, however, for the safety of the animal, that the shell should be occasionally closed, it is furnished with strong muscular bands, which pass directly from one valve to the other. When this muscular apparatus contracts, the shell is closed with considerable force. An elastic ligament of peculiar structure, is also united with the muscle, and attached to the different sides of the shell, in order to prevent it from opening beyond a certain extent.

Some bivalves, as the *Cardium*, are furnished with a kind of leg, which they extend from between the edges of the shell, and which they use as an instrument of motion. It also enables them to burrow in the sand, either to seek for some article of food, or to conceal themselves from the attack of an invading foe. This process is undoubtedly, to a great extent, muscular, and although the animal is acephalous, it must have a kind of nervous endowment, otherwise it would be unable either to feel the sand in which it burrows, or to perceive the approach of danger.

But the most singular of all the mechanical functions of the *Acephala*, is the facility with which the *Pinna* or Marine Muscle, inhabiting the shores of tempestuous seas, will spin a kind of thread, like that of the spider, with which it can attach itself so firmly to the neighboring rocks, that the surge may dash against it, for any length of time, without being able to dislodge it from its moorings. Our author observes that these threads are so strong, that the Sicilians manufacture them into gloves, and other articles resembling silk.

The next grade of life, in the Mollusca, after we leave the Acephala, is the order *Gasteropoda*, which inhabit univalve turbinated shells. The snail is of this species. Although its motion is very limited, still it moves with more facility than the preceding. Its nervous organization is better developed, as may be seen by the lengthened horns or feelers, which the animal protrudes from its head, at will, for the purpose of exploring the path along which it travels.

Shells are formed by a deposit of calcareous matter upon their surface, from the skin, or in the language of late writers, *the mantle* of the animal. The deposit is fluid in the early periods, but it soon hardens into layers more or less dense, according to the class to which the shell belongs.

Although shells are formed by a process of organization, still they are not endowed with any of the vital properties, but appear to be mere appendages for the protection of the animals. They bear the same relation to the molluscous that nails and hoofs do to terrestial animals. The formation of shells at first view, seems to bear a striking resemblance to the growth of flat bones in the Mammalia, but upon a close examination, the processes are found to be essentially different. In the former the calcareous substance exudes from the mantle, beneath the epidermis, and between that tunic and the subjacent corium, being placed beyond the reach of any vascular apparatus as soon as it escapes, while in the latter the osseous materials are deposited, in a kind of cartilaginous mould, by a regular set of vessels, which remains as a part of the organization in order to replace such portions as may be removed by mechanical or other causes.

In univalves the growth of the shell is in a spiral direction, from left to right, which is said to be the result of the location of the heart and other vital organs. In some of the marine shells, this order is reversed, and the whorls run from right to left, hence they are termed *sinistral* or reversed shells.

There are many other interesting facts, mentioned by our author, in relation to the formation of shells, which we have neither time nor space to examine. We shall also be compelled to pass by two or three families of mollusca, which present many curious and interesting phenomena, worthy the attention of the general physiologist. We shall, however, notice some of these, when we reach the department in which he examines the vital functions of the same animals.

We shall next examine our author's remarks on the *Articulata*, a tribe of animals which are said to be placed above the Mollusca, in the scale of life. We will here remark, however, that it is extremely difficult to determine, in many instances, the place the animal should occupy, for the most perfect specimens of a lower order, frequently appear to be better formed, than the most imperfect of that which is immediately above it. This is the case with the order we are about to examine — the *Articulata.*

The lowest division of articulated animals, comprehends the *Annelida*, or vermiform animals, of which the common earth-worm is a familiar example. Indeed it is questionable with us, whether this is any more complex in its structure, than the Mollusca, or indeed whether it is equally as much so as many of the most perfectly organized of the latter animals. It seems to us that a line drawn from the Zoophyte to the Vertebrated class, would entirely pass the one we are about to notice, or embrace only the most perfect specimens, as the various species of *Arachnida.*

In the Annelida the basis of the body is external, and consists of the integument that is composed of horny rings, which, when united, form a kind of cylinder in which the vital organs are located. These rings separate the body into different segments, each of which appears to be endowed with a kind of separate vitality, for the animal may be divided into different portions and still each retain a vital action.— This, however, is only the case with the lowest order of worms. All of these rings are connected by muscular attachments which move the animal by means of their various contractions. The alimentary canal, and indeed the whole of the vital organs including the circulatory, are of the most simple kind.

The next order which our author examines is termed the *Arachnida*, which embraces the different species of spiders, as well as a variety of other animals allied to them in conformation. In this animal, the rings which we have mentioned as forming the framework of the Annelida, are consolidated so as to form two principal parts of the body, the thorax and abdomen. These are separated by a deep groove which leaves only a slender tube of communication between them. The male is furnished with four pairs of legs, which are united to the thorax, while the female has five, the additional ones being for the purpose of enabling her to carry her eggs. Spiders cast off their external coverings at regular periods during their existence. Indeed this is common with all articulated animals, being the commencement of that series of metamorphosis which forms a leading trait in the winged insects. This little animal moves with great facility in various directions, and has been remarkable from time immemorial for its instincts in relation to the construction of its web and the destruction of its prey.

The *Crustacea*, the next species to which our attention is directed, possesses many singular faculties, and among them the power of reproducing, at pleasure, those parts which may have been lost or injured by mechanical causes. No sooner is an animal of this kind deprived of a claw than a growth commences upon the original stump which continues until the part is entirely restored. We have no examples of the restoration of lost parts in the higher orders of animals. In such instances the sprouting of the new claw resembles the budding of a plant. Reaumur supposed that this growth was the offspring of a concealed claw which remained within the body of the animal in the same manner that the secondary teeth are kept, for a given time, within the jaw of the child. There are, however, many objections to this hypothesis which cannot be overcome. Notwithstanding any part of the limb can be restored, yet it seems the growth is most rapid when the claw is broken off at the first joint. Indeed the animal frequently breaks it off at this point itself when it is injured near its extremity. The Crustacea, like the former family of animals, cast off their coverings, at stated periods.

We would be much pleased to examine at length, the remarks of our author on insects, but the extent of the subject will prevent us from doing more than bestowing a very cursory notice on the most remarkable phenomena in their construction.

The growth of insects is subject to a long series of modifications, in their passage through the various intermediate stages of development, before their forms are perfected. In the early periods of their existence, they are compelled to travel over the surface of the earth, or to rise above it by following the stems of plants, or other vertical structures, but in their perfect state, they can mount upon the wing and travel through the air with great velocity, collecting their food from such flowers or plants, or other sources as their instincts may point out to them. Their state of perfection, however, is of but short duration. A few summer suns and their flight is arrested, for they no sooner deposit their eggs, than their life ceases. The whole series of metamorphoses is strikingly displayed in the history of the butterfly or moth tribe. In the first instance, we have an egg, then a worm, then a kind of tropid chrysalis, and then a beautiful insect, furnished with wings, and decked in all the varied colors of animated nature. As physiologists it is only interesting for us to know, that the most imperfect state of the insect, after it leaves the egg, contains the rudiments of the various organs of its complete development.—At each period of its existence it only throws off the garment which concealed its prior form. The wings and other organs are not complete, it is true, in the *larva*, yet they are constantly increasing in size, until the *chrysalis* throws aside its mask, and proclaims, by its development, its elevated rank among the lesser inhabitants of the globe.

The muscular tissue of insects has immense power in proportion to its development. Ants will carry loads forty or fifty times the weight of their own bodies, which is a degree of strength far beyond that of the horse. Linnæus says that if the elephant had been endowed with the strength of the beetle, in proportion to its size, it would have destroyed the largest vegetable structures, and hurled rocks equal in size to the giants of ancient mythology.

We now come to the study of vertebrated animals which comprehend all the larger species on the globe, including even man himself.

Here we have an entirely different arrangement, in the organs and tissues of the body. The muscles and other soft parts surround the solid framework, instead of being placed within and covered by a horny casement. The bones of the Vertebrata, are used by the muscles as levers in performing the functions of locomotion, mastication and respiration. The most important organs, as the brain and spinal marrow, alone are placed in a bony casement.

The Vertebrata have the largest extent of motion. Their organs are not subject to the various changes of the animals we have just noticed, but are all developed in regular and harmonious succession, without the metamorphoses of the insect, or the change in covering of the Crustacea. It is true the exfoliation of the cuticle bears some resemblance to the casting of the shells and coverings of different animals, but when closely examined they will be found to be essentially different.

The composition of bone varies essentially from that of shell. The former is composed almost entirely of phosphate of lime, the particles of which are united by a force of adhesion, together with a transverse fibrous arrangement; while the latter consists principally of the carbonate, arranged in thin scales, and united by an adhesive animal matter. Bones are deposited by a process of nutrition, in the interstices of a kind of cartilaginous mould;* while shell, as has already been remarked, is

* Although this substatance is generally called cartilage in our works on anatomy and physiology, yet its composition is essentially different. Cartilage is mostly formed of albumen, while the basis of bone conststs principally of gelatine.

formed by a kind of mechanical exudation from the mantle of the molluscous animal. As the formation of bone, however, is familiar to our readers, we will not pursue the subject any further at present.

Passing by the remarks of our author on fishes, and terrestrial vertebrata, we come to that portion of his work, which treats of the *Mammalia*, or those animals which possess a spinal column, breathe air by means of lungs, and are warm blooded and viviparous, conditions which render it necessary that they should have *mammary* organs, for the support of their young. Here he traces the different tribes of animals, with their various modes of existence, and the organization necessary to enable them to procure their appropriate food, and to prepare it for the purposes of nutrition. Every animal has all in its construction that is necessary to enable it to live in its appropriate sphere, and to procure its food according to its habits and instincts. Although there is more or less uniformity of plan, in the whole, still each tribe is furnished with its proper bones and muscles, all of which are more complicated as we approach man, in whose structure we contemplate capacities, widely different from all that is below him.

Leaving what he has said in relation to the mechanical functions of birds, we shall now notice his remarks on the vital functions of organized matter.

Could the body remain continually in a state of perfection, many of those functions which are strictly vital, would be unnecessary after its growth had been completed. This, however, is not the case. Every muscular contraction produces some vital, chemical, or mechanical change, in the various parts of the body. A certain amount of nervous fluid and muscular irritability is exhausted, and a process of vitality is necessary to restore the waste. Indeed constant change appears to be one of the conditions of organized existence. The particles of which the body is composed, are constantly passing off, either as secretions or excretions, and nutrition is necessary to supply their places. This requires an extensive apparatus, which like the mechanical functions, is constantly becoming more complicated as we rise in the scale of life. The ancients supposed that a period of seven years was sufficient to work an entire revolution in the structure of the body, but it is probable that even bone itself does not retain any of its original particles, for even half that period. The process by which the system is enabled to support itself amidst this waste of materials, is termed *nutrition*, which embraces Assimilation, Circulation, Respiration, Secretion, Absorption and Excretion.

As Roget has added nothing to what was already known in relation to these functions in general, we shall not, follow him through all he has said on that subject. We shall, therefore, turn at once to his chapter on vegetable nutrition, a subject but little referred to in our works on physiology.

The older authors supposed that plants were able to live on water alone, but this doctrine has been overturned by late observations. Indeed we need not be surprised that the ancients maintained this opinion, for we have heard a distinguished teacher of medicine, of the present century, declare that plants had the power of forming carbon out of water, and molluscous animals, carbonate of lime out of nothing!

Most plants obtain their food from the soil in which they are placed. It is usually dissolved by the water which percolates through the earth, and then passes readily through the capillaries of the roots into the body of the plant, and from thence to the leaves, where it undergoes a change analagous, to that which takes place in the chyle and venous blood of the animal in its lungs.

The roots are the principal organs of absorption in vascular plants, while the *Algæ* perform the same function, by means of their external surface. The same is true, to a greater or less extent, with most of the *Fungi*. The roots of vascular plants terminate in small filaments, or *spongioles*, and it is here that absorption takes place. Some have supposed that the extremities of roots have the faculty of drawing the particles of nutritious matter from the soil, by means of a kind of suction, without being placed in actual contact with them, but this supposition is probably unfounded. The roots seem to travel in every direction in quest of food, and it is only when they reach it, that it enters the open mouths of the extreme radicles. Roget thinks that this absorption is chiefly, if not altogether mechanical, but the spongioles are no doubt endowed with a peculiar vitality, which enables them to select their food from the media in which they are placed, and to contract upon it, in order to push it onward in the vascular or cellular tissue. This vitality is probably only a modification of the organic life of Bichat, which is common to both animals and vegetables — the spongioles, like the lacteal vessels resisting the entrance of foreign agents, and thus preserving the peculiar textures of the plant, though surrounded by a foreign soil. He observes that when the spongioles are placed in a glutinous fluid, of much density, their pores are soon filled and the circulation is arrested; or when poisonous matter, as the sulphate of copper, is presented to them, in a state of solution, it is received into the plant, and its life at once destroyed. Although it would seem, from these remarks, that death was invariably the result of the application of such agents, yet repeated experiments of our own, which will be varied during the coming season, and perhaps published, lead us to doubt the truth of this assertion. We are aware that the irritability of vegetable tissues, is a subject involved in much obscurity, yet it seems evident to us, that the vitality we have already referred to, does exist, and that a plant as well as an animal, is endowed with a kind of instinct, which enables it to perform its various functions, during the periods of its existence. If a plant peculiar to dry ground, be placed in a wet soil, it will invariably send its roots towards the high land, if any be in its vicinity. The reverse is the case, if a water plant be placed in dry loam, near a marsh or pool of water.

Sap, when received into the vessels of a vegetable, rises principally through the alburnum of ligneous plants, until it reaches the leaves, where it undergoes the important alterations already mentioned. In the early periods of the development of buds, the fluid, termed by vegetable physiologists, *nursling*

*sap*, ascends through the innermost circle of wood, or that which immediately surrounds the pith, until it reaches the young bud, which at once commences a growth, that is continued until the leaves are prepared to accomplish their important functions. What change this fluid undergoes, in its passage to the bud, is entirely unknown, but it probably combines with some agent previously existing in the channels through which it passes, and by a process of elaboration, is prepared to perform its part, in the early periods of vegetable nutrition.

The channels through which the sap finds its way from the roots to the leaves, have never been ascertained. De Candolle, one of the best writers on this subject, says that it passes through the intercellular spaces, but although he adduces many facts in favor of his position, the subject is by no means settled.

The power which accomplishes the circulation of vegetables, is also involved in much obscurity. It no doubt depends upon a kind of contractility, which has, as yet, escaped our notice. Hales ascertained that the fluids arose by a power equal to the pressure of between two and three atmospheres. The velocity of the sap varies, however, in different plants, or at different periods in the same plant.

As soon as the sap arrives at the leaves, a process of exhalation commences, which reduces the watery proportions, about two thirds. The quantity of fluids that thus escapes from the plant, is about seventeen times as great as that which passes off from an equal portion of animal matter. It is curious that heat does not exercise any thing like the influence over vegetable exhalation, which light does, and hence it is only during the day that this function is in the greatest activity.

The exhalation of plants takes place through the *stomata*, or pores in the leaf, consequently the structure of leaves controls to a great extent, the escape of fluids from their surfaces.

Besides the increase of density, which takes place in the sap, other important changes are accomplished in the leaf.—It is here that a change is produced in its relative constituents; the oxygen passing off, while the carbon is retained, a process opposite to that which is accomplished in the lungs of the animal. After the oxygen is disengaged, the carbon is left ready to be deposited wherever it is necessary.

It is in the green substance of the leaf alone that this peculiar change, which may well be termed the respiration of vegetables occurs. But the process does not stop here. As soon as the sun disappears a reversed action takes place. Oxygen is absorbed, and, uniting with the carbon which has arrived from the vessels of the plant, combines with it and forms carbonic acid. The mechanism by which this is carried on is not understood by the best physiologists. Whether it is accomplished by means of a contraction and relaxation of either the air cells or proper structure of the leaf, or whether it is the offspring of a peculiar absorption cannot readily be ascertained. We think that we have discovered cells of considerable magnitude, in the leaves of the Cucumis sativus, but their proper functions could not be determined. They are undoubtedly connected in some way or other with the function of respiration.

We must acknowledge that the necessity of the reversed action, which takes place day and night, in the leaves of plants, is not so apparent to us as it appears to be to our author. He says that "it is evident that the object of the whole process is to obtain carbon in the precise state of disintegration, to which it is reduced at the moment of its separation from carbonic acid by the action of solar light on the green substance of the leaves; for it is in this state alone that it is available in promoting the nourishment of the plant, and not in the crude condition in which it exists, when it is pumped up from the earth, along with the water which conveys it into the interior of the plant." Now it seems to us that it is only in the state of carbonic acid that the carbon is admitted into the roots of plants during the earliest periods of absorption. We know of no means by which pure carbon can be dissolved in water free from any foreign impregnation. Sir H. Davy attempted to make the roots of plants absorb the most finely comminuted carbon, but failed entirely during a long series of experiments. We must still think, therefore, that the object of this absorption of oxygen at one period of the day and elimination of it at another, is involved in obscurity, and it yet remains for some student of nature to enlighten us on the subject. It is more than probable that plants do not obtain even the principal part of their carbon from the soil. They undoubtedly get large portions of it from the atmosphere, and it seems to us quite likely that it is for this purpose that the air, which always contains some carbonic acid, is taken into the structure of the leaf at night, and the oxygen thrown off again during the day. If this be true the atmosphere is daily receiving large accessions of oxygen from the vegetable kingdom, while it is as constantly obtaining carbonic acid from combustion and animal respiration. Thus the two grand kingdoms harmonize in their effects upon each other, even while life continues; the one removing from the atmosphere that which is injurious to the other, and thus preserving the proper balance upon which the welfare of both depends.

We now come to a most interesting part of vegetable nutrition—the return of the sap.

No additions are made to the tissues of the plant, during the ascent of the sap, for it must be elaborated in the leaf before it is fit for vegetable nutrition. The entire growth is accomplished after the fluid commences its return to the inferior parts of the vegetable. Our author supposes that a large proportion of the water of the sap, is actually decomposed, and that its separate elements, oxygen and hydrogen, are combined with certain portions of carbon, hydrogen, nitrogen and various earthy metals and salts, so as to form the proximate vegetable products which are found in the returning sap.

Gum is the simplest, and generally the most abundant of all the vegetable products. It is also invariably found in the returning sap, and hence it is said to be the basis of vegetable nutriment. From the investigations of Prout, it appears that gum is composed of one atom of water, and one of carbon, or in other words, that 1000 grains of gum are composed of 586 grains of oxygen and hydrogen, in the exact proportion in which they would have united to form 586 grains of water, together with 414 of carbon, hence it would seem that this article may have been readily formed in the leaves.

The vessels through which the sap descends have given rise to some discussion among physiologists. Some maintain that it passes through a peculiar set of vessels, while others think that it finds its way through the same vessels in which it ascended to the leaves. Roget inclines to the latter opinion, but we think that, the former alone can be maintained. It seems to us absurd to contend that currents of different fluids, disposed to mix with each other, should pass in opposite directions through the same channels at the same time. Be this, however, as it may, a part of the sap descends through the liber, or innermost layer of bark, while another portion passes through the alburnum, or outermost layer of wood in exogenous plants. In its passage it deposits the materials for the future alburnum and liber, in the form of a glutinous substance, termed *cambium*, which is eventually condensed into the organization proper to the plant. The roots are elongated by the sap on its return to their extremities from the leaves, so that they increase in length after the buds are developed.

It is interesting to remark the little difference there appears to be, between the composition of pure gum, and the proximate principles of vegetables. We have already remarked that 1000 grains of Gum Arabic contains 586 of oxygen and hydrogen, united in the proportions in which they exist in water, and 414 of carbon. Now the principal vegetable products, fecula, sugar, and lignin consist of the following materials—dried starch of 560 parts of water and 440 of carbon, sugar of 572 of water and 428 of carbon, and lignin of 500 of carbon and 500 of water. Thus it would require but little change in the elements of gum to produce any of the materials above enumerated.

But there are several saline compounds, and earthy, and metalic bases, which are carried into the structure of plants. The most important of these are silicum and potassium.

Besides the productions which are conveyed into the vegetable by means of the sap, or formed from it by a simple process, such as has already been mentioned, there are others which require a more complicated apparatus, as well as higher powers of vital action, to complete the necessary changes. Such are termed vegetable secretions. They are formed in the glandular tissue of plants. It is to these organs that vegetables principally owe their medicinal properties. We should like to follow our author through all he has said on this subject, did time permit. One of the most striking of the vegetable secretions, is the caustic liquor, which is collected in the vesicles, at the base of slender hairs on the stems and leaves of the common nettle.* This fluid has recently been ascertained to be alkaline, and the mechanical process by which it is thrown into the animal tissues, which come in contact with the minute spicula, has already been mentioned. The vesicles are entirely membranous, so that the least pressure on the hairs, or hollow spicula alluded to, will cause their contents to flow into whatever their extremities are introduced.

But plants not only perform a function of secretion, for they also throw off effete or foreign matter by excretion. This they do principally by their roots, for the sap on its passage downwards, is constantly being loaded by such particles as have become useless in the vegetable economy.

We come now to make some remarks on what our author has said, on animal nutrition, and here we must necessarily be very brief. We shall only notice his observations on nutrition, in the lower classes of animals, as he has added nothing to our former stock of knowledge on the same subject, in the more perfectly developed species, and we take it for granted,

* Urtica dioica.

that our readers are already acquainted with all the doctrines of the standard writers, on this interesting subject.

The food of animals differs essentially from that of vegetables, at least in those of complex organization. Animals do not draw their subsistence directly from the soil, but on the contrary, they can live alone on that which has previously been arranged by a vital chemistry. Indeed the principal, if not the only part which vegetables are destined to perform in animated nature, is the formation of materials which are to supply subsistence to a higher department of life. The principal chemical changes which take place in vegetable products, in their passage into animal structures, is the abstraction of carbon and the addition of nitrogen. But all animals do not subsist on vegetables, for whole tribes live on those which are either below or above them in the scale of life. Some live on insects, while others consume the carcases of larger animals. Indeed so great is the appetite for animal food, that when an ox is slain, all that is left of him by man will immediately be devoured by rapacious birds, quadrupeds and insects. "But," says our author, "if we follow the undecomposed particles of animal matter, which may have been swept down by the rains, and deposited in pools and lakes, amidst waters collected from the soil on every side, we here find them under favorable circumstances again partaking of animation, and invested with the various forms of infusory animalcules, which sport in countless myriads, their ephemeral existence, within the ample regions of every single drop." It seems, however, that they do not stop here, for they soon float along the rivers into the sea, where they contribute to maintain the innumerable hosts of animated beings, which people every portion of the ocean, and which rise in the regular gradation, we mentioned in the early part of this review, from the microscopic monad and scarcely visible medusa, through endless tribes of mollusca, and of fishes up to the huge leviathan of the deep. Some suppose that even the minute particles of animal matter, which are not sufficiently large to remain on the surface of the earth, rise into the atmosphere, where they support an innumerable family of animalcules, giving rise to a great variety of diseases that are the means of reducing the human frame to atoms, which again take the never ending cycle of vicissitudes and transmutations.

In his next chapter, he notices the nutrition of the lowest orders of animals. Those which belong to the polypi, present us with the simplest form of the organs of nutrition. Here nothing is to be found but a kind of stomach, consisting of a simple sack or tube, for the reception and digestion of food. We cannot trace the rudiments of a brain, heart, lungs or vessels of any sort. The body seems to consist of a pulpy mass, hence it is probable that the only way in which the aliment enters the system after the food is digested, is by a kind of capillary imbibition.

The hydra, a species of polypus, though unable to move from the rock on which it is placed, devours all kinds of animal food that comes within its reach. A great number of tentacula, or small filiform processes, surround its head extending in every direction, for the purpose of arresting the food that may come within its reach, and conveying it into the mouth of the animal. Even small fish are thus caught and devoured with the greatest eagerness.

It has been ascertained, by Trembley, that a hydra may be completely turned inside out, like the finger of a glove, and still perform all its former functions, after the external skin has been converted into a digestive apparatus.

It is also proved that the same animal may be divided longitudinally into a great many different pieces, and still each survive the injury and become a separate head, having the various organs which belonged to the original. Different polypi may be engrafted upon a single body or a number of bodies may be united, by removing a portion of their external surfaces, and thus a compound monster may be produced, equally complicated with the boasted forms of ancient fable.

Although the infusoria cannot be seen by the naked eye, still they have their appropriate food, which is received into a digestive apparatus, where it undergoes all the changes necessary for the nutrition of the animal.

The digestive organs in all the lowest animals, are extremely simple, mostly consisting of a number of sacks, without any anal terminations. Such is the case with the animals last named. Ehrenberg counted over two hundred stomachs in a microscopic monad, where the same organ seemed to perform the functions of digestion, nutrition, and circulation.

As we rise in the animal scale, however, this extreme simplicity is exchanged for complicated apparatus. We soon find a heart and vascular system, as well as a stomach and alimentary canal. The labor is divided among different organs, and consequently it is more perfectly performed.

We should like to follow our author through all his investigations in comparative anatomy, in relation to the organs of prehension and mastication of food, but as this review has already extended beyond the limits assigned to it, we must hasten to a close. Suffice it to say, that the teeth of animals always bear a direct relation to the food upon which they subsist. So well was the great Cuvier assured of this fact, that he did not hesitate to assign an animal its proper place, and to infer its various habits and instincts, from the examination of a single tooth.

In relation to the digestive apparatus it, too, is modified by the habits of animals, and the nature of their food. Those which live on vegetable diet, have alimentary canals, much longer and more complicated than those that subsist on a different kind of food.

The circulation of the lower classes of animals is a subject of much interest, in as much as it shows a general gradation of organs, always increasing in complexity, as we ascend the scale of life. In Zoophytes and the Infusoria, with other simple classes, the contents of the alimentary cavities pass into the system of different animals by means of a kind of mechanical imbibition. In the Medusa the circulation appears to be cellular, but in the Asteriæ, a grade but little above the last named, the rudiments of a vascular structure, according to Tiedemann, is observable. Here, however, it communicates with an alimentary canal and respiratory organs, and of course, there can be no regular circulation of fluids.

Insects have a kind of mixed circulation; the fluids being first placed in the dorsal vessels, and then diffused in the cellular structure, they continue to move onwards, propelled by a contraction of the vessels and cells, until the circulation though simple is complete.

When we reach the Mollusca we find the vascular system changing from a mere dorsal vessel, to a smaller centre, with regular vessels proceeding from it. When we arrive at the Lobster or Crab it is changed into an oval or globular organ, with regular contractions sending its contents through a system of vessels to all parts of the body. This may be termed the heart of the animal, although its structure is much more simple than the same organ in the warm blooded animals.—As we advance still higher in the scale, this organ changes from a mere vessicle to a compound instrument having different cavities and vessels.

Our author seems to think that the blood in the mammalia passes directly from the capillaries of the arteries into the capillaries of the veins, but this view of the subject is rather behind the improvements of the day. The blood instead of passing directly from one set of vessels to the other is thrown into a kind of areolous tissue, where it moves in various directions until nutrition is accomplished. Indeed he seems in many instances, to adhere to the older doctrines in the minutiæ of the science.

In the higher classes of animals, and where the circulation is most perfect, a set of vessels carries the blood to the organs of respiration, while another returns it to the heart, to be distributed throughout the general system, but in the lowest, one set of vessels or cells, performs both the functions of respiration and nutrition. The blood instead of being presented to the air cells of the lungs, is distributed to the external parts of the body where it meets with a sufficient quantity of air to effect the necessary changes.

In the Crustacea, the veins convey the blood to the vessels of the gills, where they enlarge into cells of considerable magnitude, in order to allow it to remain for sometime, in contact with the air of the water. After it has undergone the necessary change it is conveyed back to the dorsal vessels, already described, to be distributed over the remainder of the body.

In fishes the internal organs of circulation consist of four cavities, opening into each other, but the heart proper belongs entirely to the gills; the systemic circulation being carried on by means of an arterial trunk. The cavities are, first, a venous sack, or opening into an auricle; second, an auricle, third, a ventricle, and fourth, a dilitation of the branchial artery, termed *bulbus arteriosus*. In reptiles the heart consists alone of an auricle and a ventricle. The lesser circulation is only a part of the greater, hence the blood is always only partially changed.

We shall pass the observations of our author on the pulmonary circulation of the highest class of animals, without comment, and proceed to notice his remarks on respiration. This is divided into different modes according to the medium the animal inhabits, and hence it is either aquatic or atmospheric. The former is either cutaneous or branchial, and the latter tracheal or pulmonary.

Zoophytes have no organs of respiration. This function is probably performed either by the external parts of the body, or by the channels through which the currents of water, already mentioned, pass. The first trace of respiratory organs with which we meet, is in the most highly organized species of Annelida, as in the *Lumbricus marinus*, or lob-worm, where the respiratory apparatus consists of two rows of branchial tufts, projecting like the plume of a feather, from each side. In the Lumbricus terrestris, or common earth-worm, there is a single row of apertures, extending along the back which open into a respiratory vesicle, situated between the integument and intestine.

In fishes the respiratory organs are more complicated.—Here they consist, of gills or *branchia*, situated on each side of the throat, and so arranged that a kind of respiratory movement will always place them in contact with a supply of water, containing the necessary quantity of air.

Aquatic insects receive the water containing air into the cavities of the body, where it remains until the air is extracted, when it is thrown into a different part of the system, in order to prepare the way for a fresh supply.

In the mollusca which breathe air, as the snail, there is a round aperture near the head into which the atmosphere is received. This leads to a membranous sack overspread with a delicate network of vessels, which seems to be the first approach towards the pulmonary organs, termed lungs in vertebrated terrestrial animals. With the structure of these our readers are already acquainted, yet we cannot resist the temptation to lay before them the evolutions which take place in the respiratory apparatus of a frog, from the tadpole until the period of perfect development. No less than three sets of organs are provided for this animal at various periods of its existence. In the first place it breathes by gills, which project in an arborescent form from the sides of the neck, but as soon as another set is formed within the head these are removed. The tadpole now respires in the same way that fishes of the ordinary structure do, but when the body is sufficiently perfect to leave the water it is furnished with lungs ready to commence the process of an elevated respiration.

On the subject of secretion our author appears to be of the non-committal school, and cannot decide whether the glands act as a kind of chemical apparatus, forming the various secretions from the blood sent to them, by a different combination of its remote or proximate elements, or whether the same tissues act as strainers separating the products spontaneously formed from the circulating mass. He appears, however, inclined to the latter opinion.

In relation to the nervous power, he assumes the positions and maintains the doctrines advanced by Wilson Philip, in his work on the Vital Functions, a book which has been studied, we hope, by all of our readers.

He supports the doctrine, that the various systems of nerves are so constructed that each is affected alone by its appropriate stimulus. The eye alone can see, and the ear hear, hence those persons who pretend to hear with the stomach or to see through opaque bodies, or with their eyes closed, are nothing less than arrant impostors. It seems to us that they are about equal to the fabulous stories, of the metalic tractors, the sympathetic powder of Digby, or more recently, the fabulous tales, of some of our eastern periodicals, in relation to the wonderful effects of what they term animal magnetism.

Of tact or touch, he says that the lower animals, as insects, use it as a kind of language universal to the species. Bees execute their various works in the hive, as well as communicate to each other the passing events in which they are interested, by crossing their antenna, and striking against each other in different ways. The same is the case with ants.

To trace the olfactory, auditory, and visual senses, through the various grades of animal development, forms one of the most interesting subjects in Physiology. This we may do in a separate article in a future number but cannot undertake it at present.

None of the lowest tribes of Zoophytes, such as sponges, polypi, and medusæ, have the least trace of a nervous system. Still they can recognise the presence of their appropriate food when placed in contact with them. This, however, must be the result of an instinct which is placed beyond the narrow bounds of our comprehension.

The first trace of a nervous system in the lowest animals is found in some of the best developed species of entozoa, but it is not until we arrive at the crustacea that it appears to be distinctly perceptible, and even here it consists of a simple longitudinal cord, with a few transverse branches.

In the vertebrated animals, the nervous system is much better developed, and consists of a spinal marrow and brain, each of which becomes more complicated as we rise in the scale of life.

As all organized bodies eventually fall into entire decay, and their structures return to their original elements, a process of reproduction is necessary to preserve the various races of animals and plants from utter annihilation. This is performed in various ways. In some species there is a spontaneous division of the body of the parent, as may be seen in Monads and Verticella.

In plants, reproduction is mostly effected by seeds which contain *germs* or imperceptible organized atoms, which enlarge under favorable circumstances until the vegetable is perfected. This is termed, *Gemmiparous reproduction.*

We have already mentioned that the Sporifera including the Hydra, and other similar animals, propagated their species by means of buds or ova thrown off from the body of the parent.

The multiplication of the Infusoria has given rise to much discussion among physiologists—some follow the views of Buffon, who regarded them as the product of an inherent power belonging to a certain class of material particles, while others contend that no organized being can spring into existence without being derived from a similar parent. The latter opinion is no doubt correct, hence we must view the infusoria as the offspring of an animal resembling themselves, in forms, instincts, and habits.

The higher classes of animals are either oviparous, or viviparous, or both, as the viper, but as the habits of these animals, in their modes of reproduction are familiar to our readers, we shall not pursue the subject any further.

In the lengthened notice we have taken of the work of our author, we have attempted to lay his views on the physiology of the lower animals, before our readers, in as condensed a form as possible, and hence we have not quoted his language, but used our own, however much our readers may thus lose in regard to elegance. The truth is the work is frequently verbose, though the elegancy of the style is sufficient to compensate for the time that may be spent in its perusal. Wherever we have differed from him in opinion, we have given our views in such manner that the reader will at once distinguish them from those of our author.

We do not wish to criticise the Bridgewater Treatises, yet it seems to us that so far as they have fallen under our notice they do not equal the expectations of the public as fully as was anticipated. The work we have noticed is among the best, but is it not probable that one equally elegent in language, and more profound and original in matter, might have been produced had those who had the charge of the funds merely offered the money paid Roget, as a premium for the best production on the department stated ? In this instance the writer would have acted under a stimulus, which would have produced greater results than a mere contract for a certain sum. Notwithstanding the lore of an eastern hemisphere, we do not hesitate to say, that twenty treatises of equal, if not superior merit, to the one we have just noticed, would have been written under the circumstances we have named, by American writers. W. W.

